# Supplementary material for: EMT- and stroma-related gene expression and resistance to PD-1 blockade in urothelial cancer
Source: Nat Commun. 2018 Aug 29;9:3503. doi: 10.1038/s41467-018-05992-x (PMC6115401; doi:10.1038/s41467-018-05992-x)
Supplement: Supplementary file 1 — Supplementary Information [file 41467_2018_5992_MOESM1_ESM.pdf]

Supplementary Information

EMT- and stroma-related gene expression and resistance to PD-1 blockade in urothelial cancer

Wang et al.

Supplementary Note 1

ITA based on different sets of T-cell markers

We compared ITA estimations based on different sets of T-cell markers. Supplementary **Fig. 11** shows the correlation of the estimated ITA using the 144 gene set versus smaller T-cell gene sets. The 144-gene estimation of ITA was highly correlated with CD3 (average of CD3D, CD3E and CD3) expression (Spearman's CC=0.94), and CD8 (average of CD8A and CD8B) expression (Spearman's CC=0.83). When the 144 T-cell markers were further filtered to retain 64 genes that were significantly overexpressed in hematopoietic and lymphoid cell lines as compared to UC cell lines in the Cancer Cell Line Encyclopedia (CCLE)[1] (adjusted p-value<0.05), the estimated ITA calculated using both gene sets remained highly correlated (Spearman's CC=0.98).

EMT score based on different EMT gene lists

To assess how different existing EMT gene signatures might impact our findings, we downloaded four EMT signature lists from two previous studies: pan-EMT signature by Mak MP et al[2] (referred to as EMT_pancancer_Mak), generic-EMT signature by Tan et al[3] (referred to as EMT_generic_Tan), bladder cancer-specific EMT signature by Tan et al[3] (referred to as EMT_BLCA_Tan) and EMT signature derived from cancer cell lines by Tan et al[3] (referred to as EMT_cellline_Tan). Note that the full BLCA-specific EMT list is not available from Tan's paper[3], hence we used a subset of generic-EMT signature genes which the author annotated as belonging to BLCA-specific list. In contrast to the hallmark_EMT genes set which contain mostly mesenchymal markers, these four EMT gene lists contain both mesenchymal and epithelial markers. The EMT score was calculated by mean expression difference between mesenchymal and epithelial markers, as described in the study of Mak MP et al[2]. As shown in Supplementary **Fig. 1**, the EMT scores obtained using different EMT gene lists highly correlate with each other (Spearman's correlation >0.8). In addition, the relationship between these different EMT scores and ITA and between the different EMT scores and purity, were highly consistent with our results using the Hallmark_EMT gene set (Data not shown). Thus, our observations were unlikely biased by the use of a particular EMT gene set.

Infiltrated cell abundance of different immune cells and survival

To estimate the cell abundance of different types of immune cells, cell markers for particular cell types were obtained using the CIBERSORT signature matrix (gene-wise standardized expression value > 2 in that cell type). The cell abundance of a particular cell type was then estimated as arithmetic mean of expression levels (in log2 scale) of markers for that particular cell type. Cox Proportional Hazards (PH) regression models were used to assess the dependence of overall survival on each immune cell abundance while controlling on EMT expression level: Surv(time, event) ~ immune cell $type_i$+ EMT. The Hazard Ratio (HR) and Wald's test p-value were

reported in supplementary **Table 1**. The HR was scaled as described in the methods of main manuscript. BH method[4] was used for correction of multiple testing.

EMT, ITA and survival in other cancer types

We further investigated the relationship between EMT, ITA and survival in other types of solid cancers. We considered a total of 24 different cancer types (subtypes) in TCGA. For each cancer type, RNAseq gene expression data ("Level_3_RSEM_genes_normalized") and patient survival data were downloaded from Firehose (2016_01_28) at the Broad Institute (https://confluence.broadinstitute.org/display/GDAC/Home/).

Similar to the case of UC, significant correlations were observed between ITA and EMT in the pan-cancer analysis, and the correlation was greatly reduced conditioning on the purity estimated by ESTIMATE and others (Supplementary **Fig. 5**). This indicates a universally existing EMT-ITA association across different types of cancer. However, like in the case of the bladder cancer, EMT signals show better correlation with survival than the stromal_ESTIMATE gene signature in most cancer types (Supplementary **Fig. 6A**). Similarly, ITA show better correlation with survival than immune_ESTIMATE gene signature which included all types of immune cells (Supplementary **Fig. 6C**).

EMT and ITA in general have disparate effects in survival across cancer types. Like in the case of UC, considering EMT and ITA together in a bivariate Cox regression model (Supplementary **Fig. 6BD**) or combining them in the form of the ITA to EMT ratio (Supplementary **Fig. 6E**) helped better reveal their prognostic effects. However, cancer-type specific distinctions may exist as observed in the glioblastoma (GBM, LGG and GBMLGG) and renal cancers (KIPAN and KIRC) datasets. Given the limited follow-up time and clinical data, and heterogeneity of treatments, in TCGA datasets and other caveats, further investigations of potential cancer-type specific differences in the prognostic significance of EMT and ITA are required.

Supplementary Tables

Supplementary Table 1 Correlation of infiltrated immune cell abundance with overall survival in TCGA bladder cancer dataset

cellName	HR	logHR	lo.logHR	up.logHR	z	Pr(>|z|)	adjustedP
NK.cells.resting	0.55	-0.59	-0.85	-0.33	-4.41	1.0E-05	1.2E-04
T.cells.gamma.delta	0.61	-0.49	-0.71	-0.27	-4.38	1.2E-05	1.2E-04
T.cells.CD4.memory.resting	0.52	-0.65	-0.95	-0.35	-4.30	1.7E-05	1.2E-04
ITA	0.56	-0.57	-0.84	-0.31	-4.26	2.0E-05	1.2E-04
T.cells.CD8	0.60	-0.51	-0.76	-0.27	-4.17	3.0E-05	1.4E-04
T.cells.follicular.helper	0.59	-0.53	-0.79	-0.28	-4.10	4.1E-05	1.6E-04
Plasma.cells	0.60	-0.51	-0.76	-0.26	-3.99	6.5E-05	2.1E-04
NK.cells.activated	0.60	-0.52	-0.78	-0.25	-3.82	1.3E-04	3.9E-04
T.cells.CD4.naive	0.62	-0.48	-0.74	-0.23	-3.72	2.0E-04	5.2E-04
Macrophages.M2	0.53	-0.64	-0.99	-0.30	-3.65	2.6E-04	5.9E-04
Dendritic.cells.activated	0.58	-0.55	-0.85	-0.25	-3.58	3.5E-04	7.2E-04
Dendritic.cells.resting	0.54	-0.62	-0.96	-0.28	-3.53	4.1E-04	7.8E-04
T.cells.regulatory..Tregs.	0.62	-0.48	-0.75	-0.21	-3.50	4.6E-04	7.9E-04
T.cells.CD4.memory.activated	0.64	-0.45	-0.70	-0.20	-3.49	4.8E-04	7.9E-04
Eosinophils	0.56	-0.59	-0.92	-0.25	-3.46	5.3E-04	8.2E-04
Monocytes	0.54	-0.62	-0.97	-0.27	-3.44	5.8E-04	8.4E-04
Macrophages.M1	0.59	-0.52	-0.82	-0.22	-3.39	6.9E-04	9.4E-04
B.cells.memory	0.68	-0.38	-0.64	-0.12	-2.87	4.1E-03	5.3E-03
Macrophages.M0	0.57	-0.57	-0.97	-0.16	-2.76	5.8E-03	7.0E-03
B.cells.naive	0.75	-0.29	-0.53	-0.05	-2.33	2.0E-02	2.3E-02
Neutrophils	0.73	-0.32	-0.61	-0.03	-2.15	3.2E-02	3.5E-02
Mast.cells.activated	0.77	-0.26	-0.58	0.06	-1.62	1.0E-01	1.1E-01
Mast.cells.resting	0.95	-0.06	-0.33	0.22	-0.39	7.0E-01	7.0E-01

Supplementary Table 2 Correlation of individual EMT and stromal markers with overall survival in TCGA Bladder dataset.

Gene	HR	logHR	lo.logHR	up.logHR	z	Pr(>|z|)	adjustedP	source
EMP3	2.18	0.78	0.51	1.04	5.79	7.2E-09	2.0E-06	EMT_marker
PTGER3	2.01	0.70	0.46	0.94	5.68	1.4E-08	2.0E-06	stromal_marker
CALU	1.88	0.63	0.41	0.85	5.62	1.9E-08	2.0E-06	EMT_marker
VIM	1.97	0.68	0.43	0.92	5.48	4.2E-08	2.9E-06	EMT_marker
LAMA2	1.82	0.60	0.38	0.81	5.46	4.6E-08	2.9E-06	EMT_marker
ECM1	1.63	0.49	0.31	0.67	5.42	6.1E-08	3.0E-06	EMT_marker
SERPINE2	1.85	0.61	0.39	0.84	5.40	6.8E-08	3.0E-06	EMT_marker
FN1	2.15	0.76	0.49	1.04	5.37	7.7E-08	3.0E-06	EMT_marker
IGF1	1.89	0.64	0.40	0.87	5.34	9.5E-08	3.3E-06	stromal_marker
LRP1	1.68	0.52	0.33	0.71	5.28	1.3E-07	3.9E-06	EMT_marker
FBN1	2.18	0.78	0.49	1.07	5.27	1.4E-07	3.9E-06	EMT_marker
CALD1	1.69	0.52	0.33	0.72	5.24	1.6E-07	4.2E-06	EMT_marker
COL6A2	1.95	0.67	0.42	0.92	5.21	1.9E-07	4.5E-06	EMT_marker
FOXC2	1.47	0.39	0.24	0.54	5.11	3.3E-07	7.4E-06	EMT_marker
TNC	1.92	0.65	0.40	0.90	5.08	3.7E-07	7.8E-06	EMT_marker
MFAP5	2.10	0.74	0.45	1.03	5.05	4.4E-07	8.6E-06	both
COL6A3	2.01	0.70	0.43	0.97	5.01	5.3E-07	9.8E-06	both
CTHRC1	2.04	0.71	0.43	1.00	4.91	9.1E-07	1.5E-05	EMT_marker
LOX	1.98	0.68	0.41	0.95	4.91	9.2E-07	1.5E-05	EMT_marker
FLNA	1.72	0.54	0.33	0.76	4.90	9.8E-07	1.5E-05	EMT_marker
F13A1	1.86	0.62	0.37	0.87	4.88	1.0E-06	1.5E-05	stromal_marker
SLIT2	1.77	0.57	0.34	0.80	4.87	1.1E-06	1.5E-05	EMT_marker
COL14A1	1.70	0.53	0.32	0.75	4.87	1.1E-06	1.5E-05	stromal_marker
SGCB	1.61	0.48	0.28	0.67	4.85	1.2E-06	1.6E-05	EMT_marker
PDGFRB	1.87	0.63	0.37	0.88	4.85	1.3E-06	1.6E-05	both
WIPF1	2.29	0.83	0.49	1.16	4.83	1.3E-06	1.6E-05	EMT_marker
PRKG1	1.61	0.47	0.28	0.67	4.83	1.3E-06	1.6E-05	stromal_marker
DPYSL3	1.72	0.54	0.32	0.76	4.82	1.4E-06	1.6E-05	EMT_marker
SGCD	1.77	0.57	0.34	0.80	4.79	1.7E-06	1.8E-05	both
PRRX1	1.99	0.69	0.41	0.97	4.78	1.7E-06	1.8E-05	EMT_marker
PTGIS	1.86	0.62	0.37	0.88	4.75	2.1E-06	2.0E-05	stromal_marker
CTGF	1.73	0.55	0.32	0.78	4.74	2.1E-06	2.0E-05	EMT_marker
TPM1	1.58	0.46	0.27	0.65	4.74	2.2E-06	2.0E-05	EMT_marker
DCN	1.87	0.63	0.37	0.89	4.72	2.3E-06	2.1E-05	both
CILP	1.84	0.61	0.36	0.87	4.69	2.8E-06	2.5E-05	stromal_marker

ZEB2	1.77	0.57	0.33	0.81	4.68	2.8E-06	2.5E-05	stromal_marker
COL5A1	1.87	0.63	0.36	0.89	4.66	3.1E-06	2.6E-05	EMT_marker
COL1A2	1.86	0.62	0.36	0.88	4.66	3.2E-06	2.6E-05	both
ITGA5	1.68	0.52	0.30	0.74	4.65	3.3E-06	2.7E-05	EMT_marker
BTK	2.40	0.87	0.50	1.25	4.62	3.9E-06	3.0E-05	stromal_marker
COL5A2	1.76	0.56	0.32	0.80	4.56	5.1E-06	3.8E-05	EMT_marker
FPR1	2.01	0.70	0.40	1.00	4.56	5.1E-06	3.8E-05	stromal_marker
EDNRA	1.72	0.54	0.31	0.78	4.56	5.2E-06	3.8E-05	stromal_marker
ISLR	1.77	0.57	0.33	0.82	4.54	5.6E-06	4.0E-05	stromal_marker
FERMT2	1.65	0.50	0.28	0.72	4.53	5.8E-06	4.0E-05	EMT_marker
MYLK	1.49	0.40	0.23	0.57	4.52	6.1E-06	4.2E-05	EMT_marker
C1QB	2.21	0.79	0.45	1.14	4.51	6.5E-06	4.4E-05	stromal_marker
LRRC32	1.67	0.51	0.29	0.74	4.50	6.8E-06	4.4E-05	stromal_marker
NID2	1.73	0.55	0.31	0.79	4.50	6.9E-06	4.4E-05	EMT_marker
CXCL12	1.74	0.55	0.31	0.79	4.49	7.1E-06	4.5E-05	both
PCOLCE2	1.64	0.49	0.28	0.71	4.49	7.2E-06	4.5E-05	EMT_marker
MS4A4A	2.05	0.72	0.40	1.03	4.48	7.5E-06	4.5E-05	stromal_marker
ZFPM2	1.67	0.51	0.29	0.74	4.47	8.0E-06	4.6E-05	stromal_marker
COL4A2	1.59	0.46	0.26	0.67	4.47	8.0E-06	4.6E-05	EMT_marker
GEM	1.68	0.52	0.29	0.75	4.47	8.0E-06	4.6E-05	EMT_marker
LGALS1	1.68	0.52	0.29	0.75	4.44	9.0E-06	5.0E-05	EMT_marker
DDR2	1.65	0.50	0.28	0.73	4.43	9.3E-06	5.1E-05	stromal_marker
CYR61	1.64	0.50	0.28	0.72	4.43	9.4E-06	5.1E-05	EMT_marker
CD163	2.07	0.73	0.41	1.05	4.42	9.8E-06	5.2E-05	stromal_marker
VSIG4	2.00	0.70	0.38	1.01	4.34	1.4E-05	7.3E-05	stromal_marker
FSTL1	1.73	0.55	0.30	0.79	4.33	1.5E-05	7.8E-05	EMT_marker
KCNJ8	1.70	0.53	0.29	0.78	4.32	1.6E-05	7.9E-05	stromal_marker
APLP1	1.44	0.37	0.20	0.53	4.30	1.7E-05	8.3E-05	EMT_marker
CDH11	1.79	0.58	0.32	0.85	4.30	1.7E-05	8.3E-05	EMT_marker
HGF	1.56	0.44	0.24	0.65	4.30	1.7E-05	8.3E-05	stromal_marker
COL3A1	1.80	0.59	0.32	0.86	4.29	1.8E-05	8.4E-05	both
COL5A3	1.65	0.50	0.27	0.73	4.27	1.9E-05	9.1E-05	both
COL1A1	1.73	0.55	0.30	0.80	4.25	2.1E-05	9.7E-05	EMT_marker
COL16A1	1.82	0.60	0.32	0.88	4.24	2.3E-05	1.0E-04	EMT_marker
SIGLEC1	2.13	0.75	0.40	1.11	4.22	2.5E-05	1.1E-04	stromal_marker
VEGFA	0.61	-0.50	-0.73	-0.26	-4.20	2.6E-05	1.2E-04	EMT_marker
GPR124	1.59	0.47	0.25	0.68	4.17	3.0E-05	1.3E-04	stromal_marker
SPON2	1.67	0.51	0.27	0.75	4.16	3.2E-05	1.4E-04	stromal_marker
ACTA2	1.54	0.43	0.23	0.64	4.15	3.3E-05	1.4E-04	EMT_marker
CD248	1.60	0.47	0.25	0.70	4.14	3.5E-05	1.5E-04	stromal_marker
SFRP4	1.83	0.61	0.32	0.89	4.13	3.6E-05	1.5E-04	both

COL8A2	1.66	0.51	0.27	0.75	4.13	3.6E-05	1.5E-04	both
SPARC	1.64	0.50	0.26	0.73	4.12	3.7E-05	1.5E-04	EMT_marker
ECM2	1.65	0.50	0.26	0.74	4.12	3.8E-05	1.5E-04	both
THBS1	1.59	0.46	0.24	0.69	4.11	4.0E-05	1.6E-04	EMT_marker
TGFBI	1.60	0.47	0.25	0.70	4.10	4.1E-05	1.6E-04	EMT_marker
C1QA	2.08	0.73	0.38	1.08	4.10	4.1E-05	1.6E-04	stromal_marker
ELN	1.65	0.50	0.26	0.74	4.08	4.4E-05	1.7E-04	EMT_marker
POSTN	1.72	0.54	0.28	0.80	4.07	4.6E-05	1.7E-04	EMT_marker
OLFML1	1.64	0.49	0.26	0.73	4.07	4.7E-05	1.7E-04	stromal_marker
TAGLN	1.68	0.52	0.27	0.77	4.05	5.1E-05	1.9E-04	EMT_marker
GAS1	1.80	0.59	0.30	0.87	4.05	5.2E-05	1.9E-04	EMT_marker
RUNX1T1	1.49	0.40	0.21	0.59	4.04	5.3E-05	1.9E-04	stromal_marker
MSX1	1.43	0.36	0.18	0.54	3.97	7.1E-05	2.5E-04	EMT_marker
PTX3	1.53	0.42	0.21	0.63	3.96	7.4E-05	2.6E-04	EMT_marker
COMP	1.77	0.57	0.29	0.85	3.96	7.5E-05	2.6E-04	both
HTRA1	1.58	0.46	0.23	0.69	3.93	8.3E-05	2.8E-04	EMT_marker
FAP	1.79	0.58	0.29	0.87	3.93	8.4E-05	2.8E-04	both
FMOD	1.66	0.51	0.25	0.76	3.93	8.6E-05	2.9E-04	EMT_marker
MS4A6A	1.99	0.69	0.34	1.03	3.92	8.9E-05	2.9E-04	stromal_marker
GLT25D1	1.64	0.50	0.25	0.75	3.92	8.9E-05	2.9E-04	EMT_marker
SFRP1	1.66	0.51	0.25	0.76	3.92	9.0E-05	2.9E-04	EMT_marker
OLFML2B	1.66	0.51	0.25	0.76	3.91	9.4E-05	3.0E-04	stromal_marker
TPM2	1.49	0.40	0.20	0.60	3.90	9.4E-05	3.0E-04	EMT_marker
CSF1R	1.84	0.61	0.30	0.91	3.90	9.6E-05	3.0E-04	stromal_marker
ABI3BP	1.64	0.50	0.25	0.75	3.89	1.0E-04	3.1E-04	EMT_marker
CAP2	1.45	0.37	0.18	0.56	3.89	1.0E-04	3.1E-04	EMT_marker
NNMT	1.76	0.56	0.28	0.85	3.87	1.1E-04	3.3E-04	EMT_marker
FCGR2A	1.89	0.64	0.31	0.96	3.85	1.2E-04	3.5E-04	stromal_marker
LUM	1.66	0.50	0.25	0.76	3.83	1.3E-04	3.8E-04	both
CD33	1.77	0.57	0.28	0.86	3.82	1.3E-04	3.9E-04	stromal_marker
BGN	1.60	0.47	0.23	0.71	3.81	1.4E-04	4.0E-04	both
WISP1	1.67	0.51	0.25	0.78	3.80	1.4E-04	4.2E-04	stromal_marker
PCOLCE	1.54	0.43	0.21	0.65	3.78	1.5E-04	4.5E-04	EMT_marker
SCG2	1.46	0.38	0.18	0.58	3.78	1.6E-04	4.5E-04	EMT_marker
C3AR1	1.91	0.65	0.31	0.98	3.76	1.7E-04	4.7E-04	stromal_marker
MSR1	1.84	0.61	0.29	0.93	3.76	1.7E-04	4.7E-04	stromal_marker
ACTG2	1.54	0.43	0.20	0.66	3.73	1.9E-04	5.3E-04	stromal_marker
LPPR4	1.40	0.34	0.16	0.52	3.71	2.1E-04	5.8E-04	stromal_marker
COL15A1	1.58	0.46	0.22	0.70	3.70	2.1E-04	5.8E-04	stromal_marker
SPON1	1.56	0.44	0.21	0.68	3.70	2.2E-04	5.8E-04	stromal_marker
MYL9	1.47	0.38	0.18	0.59	3.69	2.2E-04	5.9E-04	EMT_marker

ITGB3	1.43	0.36	0.17	0.55	3.66	2.5E-04	6.6E-04	EMT_marker
CD14	1.74	0.55	0.26	0.85	3.66	2.5E-04	6.6E-04	stromal_marker
LEPRE1	1.40	0.34	0.16	0.52	3.65	2.7E-04	6.9E-04	EMT_marker
LMOD1	1.46	0.38	0.17	0.58	3.65	2.7E-04	6.9E-04	stromal_marker
PLOD1	1.42	0.35	0.16	0.54	3.64	2.7E-04	7.0E-04	EMT_marker
COL4A1	1.43	0.36	0.16	0.55	3.62	2.9E-04	7.5E-04	EMT_marker
PMEPA1	1.47	0.39	0.18	0.60	3.62	3.0E-04	7.6E-04	EMT_marker
THY1	1.52	0.42	0.19	0.65	3.60	3.1E-04	7.9E-04	EMT_marker
ADAM12	1.64	0.49	0.23	0.76	3.60	3.2E-04	7.9E-04	both
PAPPA	1.52	0.42	0.19	0.64	3.58	3.5E-04	8.6E-04	stromal_marker
ITGAM	1.74	0.55	0.25	0.86	3.56	3.7E-04	9.1E-04	stromal_marker
ANPEP	1.35	0.30	0.13	0.46	3.54	3.9E-04	9.5E-04	EMT_marker
GREM1	1.58	0.46	0.20	0.71	3.54	3.9E-04	9.5E-04	both
EFEMP2	1.47	0.38	0.17	0.59	3.54	4.0E-04	9.6E-04	EMT_marker
INHBA	1.63	0.49	0.21	0.76	3.48	4.9E-04	1.2E-03	EMT_marker
CDH2	1.43	0.36	0.16	0.57	3.46	5.5E-04	1.3E-03	EMT_marker
VCAN	1.56	0.45	0.19	0.70	3.46	5.5E-04	1.3E-03	EMT_marker
SERPING1	1.69	0.52	0.23	0.82	3.45	5.7E-04	1.3E-03	stromal_marker
CRLF1	1.38	0.32	0.14	0.50	3.44	5.8E-04	1.3E-03	EMT_marker
HEPH	1.48	0.39	0.17	0.61	3.43	6.1E-04	1.4E-03	stromal_marker
ENPEP	1.36	0.31	0.13	0.49	3.42	6.4E-04	1.4E-03	stromal_marker
AIF1	1.80	0.59	0.25	0.93	3.41	6.4E-04	1.5E-03	stromal_marker
COX7A1	1.45	0.37	0.16	0.58	3.40	6.7E-04	1.5E-03	stromal_marker
GJA1	1.42	0.35	0.15	0.56	3.36	7.9E-04	1.8E-03	EMT_marker
FUCA1	0.71	-0.34	-0.54	-0.14	-3.34	8.2E-04	1.8E-03	EMT_marker
PIK3R5	1.91	0.65	0.27	1.03	3.33	8.7E-04	1.9E-03	stromal_marker
LOXL2	1.45	0.37	0.15	0.59	3.30	9.7E-04	2.1E-03	EMT_marker
MGP	1.45	0.37	0.15	0.59	3.30	9.7E-04	2.1E-03	EMT_marker
FGF2	1.41	0.34	0.14	0.55	3.28	1.0E-03	2.2E-03	EMT_marker
ITGBL1	1.56	0.44	0.18	0.71	3.28	1.0E-03	2.2E-03	stromal_marker
SULF1	1.56	0.44	0.18	0.71	3.28	1.1E-03	2.2E-03	stromal_marker
PLXDC1	1.43	0.36	0.14	0.57	3.26	1.1E-03	2.3E-03	stromal_marker
FBLN5	1.46	0.38	0.15	0.61	3.26	1.1E-03	2.4E-03	EMT_marker
IL6	1.52	0.42	0.17	0.68	3.25	1.2E-03	2.4E-03	EMT_marker
LAMC1	1.41	0.35	0.14	0.55	3.25	1.2E-03	2.4E-03	EMT_marker
ERG	1.40	0.33	0.13	0.53	3.24	1.2E-03	2.5E-03	stromal_marker
PDE2A	1.30	0.26	0.10	0.42	3.23	1.2E-03	2.5E-03	stromal_marker
LY86	1.59	0.47	0.18	0.75	3.21	1.3E-03	2.7E-03	stromal_marker
TNFSF4	1.53	0.43	0.17	0.69	3.21	1.3E-03	2.7E-03	stromal_marker
NOTCH2	1.36	0.31	0.12	0.50	3.20	1.4E-03	2.7E-03	EMT_marker
JUN	1.38	0.32	0.13	0.52	3.20	1.4E-03	2.8E-03	EMT_marker

FBN2	1.45	0.37	0.14	0.60	3.19	1.4E-03	2.8E-03	EMT_marker
SDC4	0.78	-0.25	-0.41	-0.10	-3.17	1.5E-03	3.0E-03	EMT_marker
SGCG	1.30	0.26	0.10	0.42	3.17	1.5E-03	3.0E-03	EMT_marker
CD93	1.43	0.36	0.14	0.58	3.17	1.5E-03	3.0E-03	stromal_marker
GADD45B	1.37	0.32	0.12	0.51	3.17	1.6E-03	3.0E-03	EMT_marker
TGM2	1.49	0.40	0.15	0.64	3.15	1.6E-03	3.1E-03	EMT_marker
CH25H	1.42	0.35	0.13	0.57	3.10	2.0E-03	3.7E-03	stromal_marker
GLIPR1	1.50	0.40	0.15	0.66	3.09	2.0E-03	3.8E-03	EMT_marker
VCAM1	1.50	0.41	0.15	0.67	3.08	2.1E-03	3.9E-03	both
FSTL3	1.42	0.35	0.13	0.57	3.07	2.2E-03	4.1E-03	EMT_marker
ITIH5	1.34	0.30	0.11	0.49	3.04	2.4E-03	4.4E-03	stromal_marker
LRRC15	1.55	0.44	0.15	0.72	3.03	2.5E-03	4.6E-03	both
TNN	1.31	0.27	0.09	0.44	3.01	2.6E-03	4.8E-03	stromal_marker
ARHGAP28	1.37	0.32	0.11	0.52	2.99	2.8E-03	5.1E-03	stromal_marker
HDC	1.39	0.33	0.11	0.55	2.96	3.1E-03	5.5E-03	stromal_marker
EDIL3	1.39	0.33	0.11	0.54	2.96	3.1E-03	5.5E-03	both
PCDH12	1.37	0.31	0.10	0.52	2.94	3.2E-03	5.8E-03	stromal_marker
LDB2	1.35	0.30	0.10	0.50	2.92	3.5E-03	6.2E-03	stromal_marker
AOC3	1.35	0.30	0.10	0.50	2.91	3.6E-03	6.4E-03	stromal_marker
PDLIM4	1.42	0.35	0.11	0.58	2.90	3.7E-03	6.5E-03	EMT_marker
RGS4	1.38	0.32	0.10	0.54	2.88	4.0E-03	7.0E-03	both
PVR	1.31	0.27	0.09	0.46	2.87	4.1E-03	7.1E-03	EMT_marker
ASPN	1.38	0.32	0.10	0.54	2.86	4.3E-03	7.4E-03	stromal_marker
PTHLH	1.31	0.27	0.08	0.46	2.85	4.4E-03	7.6E-03	EMT_marker
MMP14	1.33	0.29	0.09	0.49	2.80	5.1E-03	8.7E-03	EMT_marker
PMP22	1.40	0.34	0.10	0.57	2.80	5.1E-03	8.7E-03	EMT_marker
BMP1	1.38	0.32	0.09	0.55	2.73	6.3E-03	1.1E-02	EMT_marker
FMO1	1.55	0.44	0.12	0.76	2.72	6.5E-03	1.1E-02	stromal_marker
FCGR2B	1.48	0.39	0.11	0.67	2.70	6.9E-03	1.2E-02	stromal_marker
GPC1	1.30	0.27	0.07	0.46	2.67	7.6E-03	1.3E-02	EMT_marker
MAF	1.32	0.28	0.07	0.49	2.66	7.9E-03	1.3E-02	stromal_marker
BDNF	1.31	0.27	0.07	0.47	2.65	8.1E-03	1.3E-02	EMT_marker
MXRA5	1.32	0.28	0.07	0.49	2.63	8.6E-03	1.4E-02	both
EGFL6	0.79	-0.24	-0.41	-0.06	-2.62	8.7E-03	1.4E-02	stromal_marker
THBS2	1.41	0.34	0.08	0.60	2.59	9.6E-03	1.6E-02	both
MAGEE1	1.25	0.22	0.05	0.40	2.49	1.3E-02	2.1E-02	EMT_marker
CD200	1.29	0.26	0.05	0.46	2.48	1.3E-02	2.1E-02	stromal_marker
CCR1	1.36	0.31	0.06	0.56	2.46	1.4E-02	2.2E-02	stromal_marker
MMP2	1.34	0.29	0.06	0.53	2.44	1.5E-02	2.3E-02	EMT_marker
LAMA1	1.29	0.25	0.05	0.46	2.42	1.6E-02	2.5E-02	EMT_marker
DAB2	1.32	0.28	0.05	0.50	2.42	1.6E-02	2.5E-02	EMT_marker

NTM	1.33	0.29	0.05	0.52	2.41	1.6E-02	2.5E-02	EMT_marker
CDH5	1.29	0.25	0.04	0.46	2.38	1.7E-02	2.7E-02	stromal_marker
COL10A1	1.40	0.34	0.06	0.62	2.35	1.9E-02	2.9E-02	stromal_marker
DIO2	1.30	0.26	0.04	0.48	2.32	2.0E-02	3.1E-02	stromal_marker
CDH6	1.25	0.22	0.03	0.41	2.30	2.1E-02	3.3E-02	EMT_marker
CD44	1.29	0.26	0.04	0.48	2.30	2.1E-02	3.3E-02	EMT_marker
COL12A1	1.32	0.28	0.04	0.51	2.29	2.2E-02	3.3E-02	EMT_marker
SDC1	0.86	-0.15	-0.28	-0.02	-2.28	2.2E-02	3.4E-02	EMT_marker
FZD8	1.28	0.24	0.03	0.45	2.27	2.3E-02	3.5E-02	EMT_marker
TNFRSF12A	1.28	0.25	0.03	0.46	2.26	2.4E-02	3.6E-02	EMT_marker
FBLN2	1.32	0.27	0.04	0.51	2.25	2.4E-02	3.6E-02	both
SAMSN1	1.43	0.36	0.04	0.67	2.23	2.6E-02	3.9E-02	stromal_marker
ITGB1	1.26	0.23	0.03	0.43	2.22	2.7E-02	3.9E-02	EMT_marker
ITM2A	1.26	0.23	0.03	0.43	2.21	2.7E-02	4.0E-02	stromal_marker
ADAMTS5	1.26	0.23	0.03	0.44	2.20	2.8E-02	4.0E-02	stromal_marker
TGFB1	1.26	0.23	0.03	0.44	2.20	2.8E-02	4.1E-02	EMT_marker
PLXNC1	1.38	0.32	0.03	0.61	2.19	2.9E-02	4.2E-02	stromal_marker
SNAI2	1.28	0.25	0.02	0.47	2.17	3.0E-02	4.3E-02	EMT_marker
SERPINE1	1.28	0.24	0.02	0.47	2.16	3.1E-02	4.4E-02	EMT_marker
FOXF1	1.24	0.21	0.02	0.41	2.13	3.3E-02	4.7E-02	stromal_marker
ITGB5	1.24	0.21	0.02	0.41	2.12	3.4E-02	4.9E-02	EMT_marker
TFPI2	1.25	0.23	0.02	0.44	2.10	3.6E-02	5.1E-02	EMT_marker
GIMAP5	1.41	0.35	0.02	0.67	2.09	3.7E-02	5.2E-02	stromal_marker
NOX4	1.25	0.23	0.01	0.44	2.09	3.7E-02	5.2E-02	stromal_marker
ATP8B4	1.31	0.27	0.01	0.53	2.04	4.2E-02	5.8E-02	stromal_marker
DKK1	1.24	0.22	0.01	0.43	2.02	4.3E-02	6.0E-02	EMT_marker
RARRES2	1.29	0.26	0.01	0.51	2.01	4.5E-02	6.2E-02	stromal_marker
GPX7	1.22	0.20	0.00	0.39	2.00	4.6E-02	6.3E-02	EMT_marker
TNFRSF11B	1.20	0.18	0.00	0.35	2.00	4.6E-02	6.3E-02	EMT_marker
FASLG	0.71	-0.34	-0.69	0.01	-1.92	5.5E-02	7.6E-02	stromal_marker
SPP1	1.24	0.21	-0.01	0.43	1.91	5.6E-02	7.7E-02	EMT_marker
ID2	0.82	-0.20	-0.40	0.01	-1.90	5.8E-02	7.9E-02	EMT_marker
RAMP3	1.24	0.22	-0.01	0.45	1.83	6.7E-02	9.0E-02	stromal_marker
TPM4	1.22	0.20	-0.02	0.42	1.79	7.4E-02	1.0E-01	EMT_marker
SNTB1	1.17	0.16	-0.02	0.34	1.75	8.0E-02	1.1E-01	EMT_marker
MEST	1.17	0.16	-0.02	0.33	1.73	8.3E-02	1.1E-01	EMT_marker
SERPINH1	1.20	0.18	-0.03	0.40	1.71	8.6E-02	1.1E-01	EMT_marker
SPOCK1	1.20	0.18	-0.03	0.39	1.71	8.8E-02	1.2E-01	EMT_marker
PLOD2	1.18	0.17	-0.03	0.36	1.68	9.3E-02	1.2E-01	EMT_marker
CD86	1.34	0.29	-0.05	0.63	1.68	9.3E-02	1.2E-01	stromal_marker
MMP1	0.83	-0.19	-0.41	0.03	-1.67	9.5E-02	1.2E-01	EMT_marker

PLAUR	1.25	0.22	-0.04	0.48	1.67	9.6E-02	1.2E-01	EMT_marker
EMCN	1.18	0.17	-0.03	0.36	1.66	9.7E-02	1.3E-01	stromal_marker
COL11A1	1.25	0.22	-0.04	0.49	1.66	9.8E-02	1.3E-01	EMT_marker
TCF21	1.18	0.17	-0.03	0.37	1.64	1.0E-01	1.3E-01	stromal_marker
ITGAV	1.19	0.18	-0.04	0.39	1.63	1.0E-01	1.3E-01	EMT_marker
LAMA3	1.17	0.15	-0.04	0.34	1.58	1.1E-01	1.4E-01	EMT_marker
CXCL14	1.16	0.15	-0.04	0.33	1.54	1.2E-01	1.6E-01	stromal_marker
ODZ4	1.16	0.15	-0.04	0.34	1.52	1.3E-01	1.6E-01	stromal_marker
IGSF6	1.30	0.26	-0.08	0.60	1.52	1.3E-01	1.6E-01	stromal_marker
CADM1	1.16	0.14	-0.04	0.33	1.51	1.3E-01	1.6E-01	EMT_marker
IGFBP3	0.85	-0.16	-0.37	0.05	-1.49	1.4E-01	1.7E-01	EMT_marker
TIMP1	1.18	0.17	-0.05	0.39	1.49	1.4E-01	1.7E-01	EMT_marker
ENPP2	1.16	0.15	-0.05	0.36	1.47	1.4E-01	1.7E-01	stromal_marker
ITIH3	1.18	0.17	-0.06	0.39	1.47	1.4E-01	1.7E-01	stromal_marker
TGFBR3	0.86	-0.15	-0.35	0.05	-1.46	1.4E-01	1.8E-01	EMT_marker
IL1B	0.85	-0.16	-0.38	0.06	-1.40	1.6E-01	2.0E-01	stromal_marker
MATN3	1.16	0.15	-0.06	0.36	1.39	1.6E-01	2.0E-01	EMT_marker
AREG	1.15	0.14	-0.07	0.35	1.34	1.8E-01	2.2E-01	EMT_marker
SP140	0.79	-0.23	-0.58	0.11	-1.33	1.8E-01	2.2E-01	stromal_marker
FAS	1.17	0.16	-0.08	0.40	1.27	2.0E-01	2.5E-01	EMT_marker
TNFAIP3	1.16	0.15	-0.09	0.39	1.25	2.1E-01	2.6E-01	EMT_marker
DST	1.13	0.12	-0.08	0.32	1.19	2.4E-01	2.8E-01	EMT_marker
OXTR	1.10	0.10	-0.07	0.26	1.15	2.5E-01	3.0E-01	EMT_marker
WNT2	1.15	0.14	-0.10	0.38	1.14	2.5E-01	3.0E-01	stromal_marker
MMP3	1.16	0.15	-0.11	0.40	1.13	2.6E-01	3.1E-01	both
VEGFC	1.11	0.11	-0.08	0.29	1.12	2.6E-01	3.1E-01	EMT_marker
NT5E	1.13	0.12	-0.11	0.36	1.06	2.9E-01	3.4E-01	EMT_marker
SLC6A8	0.90	-0.10	-0.29	0.09	-1.04	3.0E-01	3.5E-01	EMT_marker
BASP1	1.11	0.11	-0.09	0.31	1.03	3.0E-01	3.5E-01	EMT_marker
TLR2	0.89	-0.12	-0.34	0.11	-1.02	3.1E-01	3.6E-01	stromal_marker
RASGRP3	0.88	-0.12	-0.37	0.12	-0.99	3.2E-01	3.7E-01	stromal_marker
TIMP3	1.11	0.11	-0.11	0.32	0.97	3.3E-01	3.8E-01	EMT_marker
CAPG	1.10	0.09	-0.10	0.29	0.97	3.3E-01	3.8E-01	EMT_marker
TFEC	1.12	0.12	-0.15	0.38	0.86	3.9E-01	4.5E-01	stromal_marker
FBLN1	0.93	-0.08	-0.26	0.11	-0.82	4.1E-01	4.7E-01	EMT_marker
TLR7	1.10	0.10	-0.14	0.34	0.79	4.3E-01	4.9E-01	stromal_marker
QSOX1	1.08	0.08	-0.12	0.28	0.76	4.5E-01	5.1E-01	EMT_marker
IL18R1	0.91	-0.09	-0.34	0.15	-0.76	4.5E-01	5.1E-01	stromal_marker
FRZB	1.07	0.07	-0.12	0.26	0.74	4.6E-01	5.2E-01	stromal_marker
IL8	1.09	0.08	-0.14	0.30	0.73	4.7E-01	5.2E-01	EMT_marker
CLEC7A	1.08	0.08	-0.16	0.32	0.67	5.0E-01	5.6E-01	stromal_marker

TXNDC3	1.07	0.07	-0.14	0.27	0.66	5.1E-01	5.7E-01	stromal_marker
CXCL1	1.08	0.08	-0.15	0.31	0.65	5.2E-01	5.7E-01	EMT_marker
KDR	1.06	0.06	-0.13	0.25	0.64	5.2E-01	5.8E-01	stromal_marker
RSAD2	1.08	0.08	-0.16	0.31	0.63	5.3E-01	5.8E-01	stromal_marker
SCUBE2	0.93	-0.07	-0.31	0.16	-0.62	5.4E-01	5.9E-01	stromal_marker
MCM7	0.95	-0.05	-0.23	0.13	-0.57	5.7E-01	6.2E-01	EMT_marker
CXCL6	1.06	0.06	-0.15	0.28	0.55	5.8E-01	6.3E-01	EMT_marker
IGFBP4	0.95	-0.05	-0.23	0.13	-0.53	5.9E-01	6.4E-01	EMT_marker
ITGA2	0.95	-0.05	-0.26	0.15	-0.49	6.2E-01	6.7E-01	EMT_marker
ABCA6	0.95	-0.05	-0.28	0.17	-0.48	6.3E-01	6.8E-01	stromal_marker
PLOD3	1.05	0.05	-0.15	0.24	0.46	6.4E-01	6.9E-01	EMT_marker
ZNF423	0.96	-0.04	-0.23	0.14	-0.46	6.4E-01	6.9E-01	stromal_marker
TRAT1	0.90	-0.10	-0.54	0.34	-0.46	6.4E-01	6.9E-01	stromal_marker
SLIT3	1.05	0.04	-0.15	0.24	0.44	6.6E-01	7.0E-01	EMT_marker
PPIB	1.04	0.04	-0.14	0.22	0.42	6.7E-01	7.2E-01	EMT_marker
SH2D1A	0.89	-0.11	-0.65	0.42	-0.42	6.8E-01	7.2E-01	stromal_marker
MATN2	1.04	0.04	-0.15	0.23	0.39	7.0E-01	7.3E-01	EMT_marker
RHOB	1.03	0.03	-0.15	0.22	0.35	7.2E-01	7.6E-01	EMT_marker
CD59	1.04	0.04	-0.17	0.24	0.35	7.3E-01	7.6E-01	EMT_marker
CXCL9	1.05	0.05	-0.33	0.42	0.24	8.1E-01	8.5E-01	stromal_marker
APBB1IP	1.02	0.02	-0.18	0.22	0.23	8.2E-01	8.5E-01	stromal_marker
PFN2	0.98	-0.02	-0.19	0.15	-0.21	8.4E-01	8.7E-01	EMT_marker
LOXL1	1.02	0.02	-0.18	0.22	0.18	8.6E-01	8.8E-01	EMT_marker
IL15	1.02	0.02	-0.23	0.28	0.18	8.6E-01	8.8E-01	EMT_marker
IL32	1.02	0.02	-0.26	0.30	0.16	8.7E-01	8.9E-01	EMT_marker
LAMC2	0.98	-0.02	-0.22	0.19	-0.16	8.7E-01	8.9E-01	EMT_marker
COPA	1.01	0.01	-0.13	0.15	0.10	9.2E-01	9.4E-01	EMT_marker
SAT1	1.01	0.01	-0.23	0.25	0.09	9.3E-01	9.4E-01	EMT_marker
IGFBP2	1.01	0.01	-0.19	0.21	0.09	9.3E-01	9.4E-01	EMT_marker
GADD45A	0.99	-0.01	-0.20	0.19	-0.08	9.4E-01	9.5E-01	EMT_marker
COL7A1	0.99	-0.01	-0.22	0.21	-0.07	9.5E-01	9.5E-01	EMT_marker
ENO2	1.00	0.00	-0.21	0.21	0.02	9.8E-01	9.8E-01	EMT_marker
WNT5A	1.00	0.00	-0.19	0.19	0.01	9.9E-01	9.9E-01	EMT_marker

Supplementary Table 3 The distribution of baseline variables in the full CheckMate 275 study and in the CheckMate 275 biomarker cohort

Variable	Full Cohort (n=270)	Biomarker Cohort (n=214)
Age, mean (min, max)	70 (38, 90)	65 (39,90)
Age ≥ 75	14%	14%
PD-L1 expression, mean (min, max)*	10.7% (0, 100)	9.4% (0, 100)
% with hemoglobin < 10 g/dL	17.8%	17.3%
% with liver metastases	28.5%	28%
% with visceral metastases	83%	83.6%
Objective response rate	20%	18.7%

*% PD-L1 expression as measured on tumor cells

Supplementary Table 4 Impact of CD8 expression and EMT/stroma_core gene expression on clinical outcomes in the CheckMate 275 biomarker cohort (n=214): test statistics

Test	Model I	Model II	Test Statistics*		
			**PFS**	**OS**	**Objective Response**
Effect of CD8_IHC alone	Intercept	Model I + CD8_IHC	14.80	8.12	10.49
Overall effect of adding EMT/Stroma_core	Intercept + CD8 IHC	Model I + EMT/Stroma_core + CD8_IHC:EMT/Stroma_core	6.23	6.71	7.66
CD8_IHC:EMT/Stroma_core interaction	Intercept + CD8 IHC + EMT/Stroma_core	Model I + CD8_IHC:EMT/Stroma_core	4.44	4.24	4.43
Effect of adding CD8_IHC to baseline variables	Intercept + HBN + LIVERMET + PDL1	Model I + CD8_IHC	15.06	8.82	12.75
Overall effect of adding EMT/Stroma_core to baseline variables and CD8_IHC	Intercept + HBN + LIVERMET + PDL1 + CD8 IHC	Model I + EMT/Stroma_core + CD8_IHC:EMT/Stroma_core	10.32	10.64	8.19
CD8_IHC:EMT/Stroma_core interaction when baseline variables included	Intercept + HBN + LIVERMET + PDL1 + CD8_IHC + EMT/Stroma_core	Model I + CD8_IHC:EMT/Stroma_core	6.82	7.94	4.67
Effect of EMT/Stroma_core alone	Intercept	Model I + EMT/Stroma_core	0.20	0.66	0.54
Overall effect of adding CD8_IHC	Intercept + EMT/Stroma_core	Model I + CD8_IHC + CD8_IHC:EMT/Stroma_core	20.82	14.17	17.61
Effect of adding EMT/Stroma_core to baseline variables	Intercept + HBN + LIVERMET + PDL1	Model I + EMT/Stroma_core	2.15	1.56	1.15
Overall effect of adding CD8_IHC to baseline variables and EMT/Stroma_Core	Intercept + HBN + LIVERMET + PDL1 + EMT/Stroma_core	Model I + CD8_IHC + CD8_IHC:EMTStroma_core	23.23	17.89	19.79

*Chi-square test statistics from likelihood-ratio hypothesis tests of the effects of CD8 IHC or EMT/Stroma_core scores on PFS, OS, and Objective Response, for the CheckMate 275 biomarker cohort. Tests for PFS and OS are from Cox PH models. Tests for objective response are from linear logistic regression models. Each test compares Model II to Model I. Degrees of freedom for each test = difference in number of terms between Model II and Model I. CD8_IHC = CD8 immunohistochemistry, EMT/Stromal_core = EMT/Stromal_core gene expression, HBN = hemoglobin; LIVERMET = presence of liver metastases; PDL1 = PDL-1 IHC score.

Supplementary Fig. 1

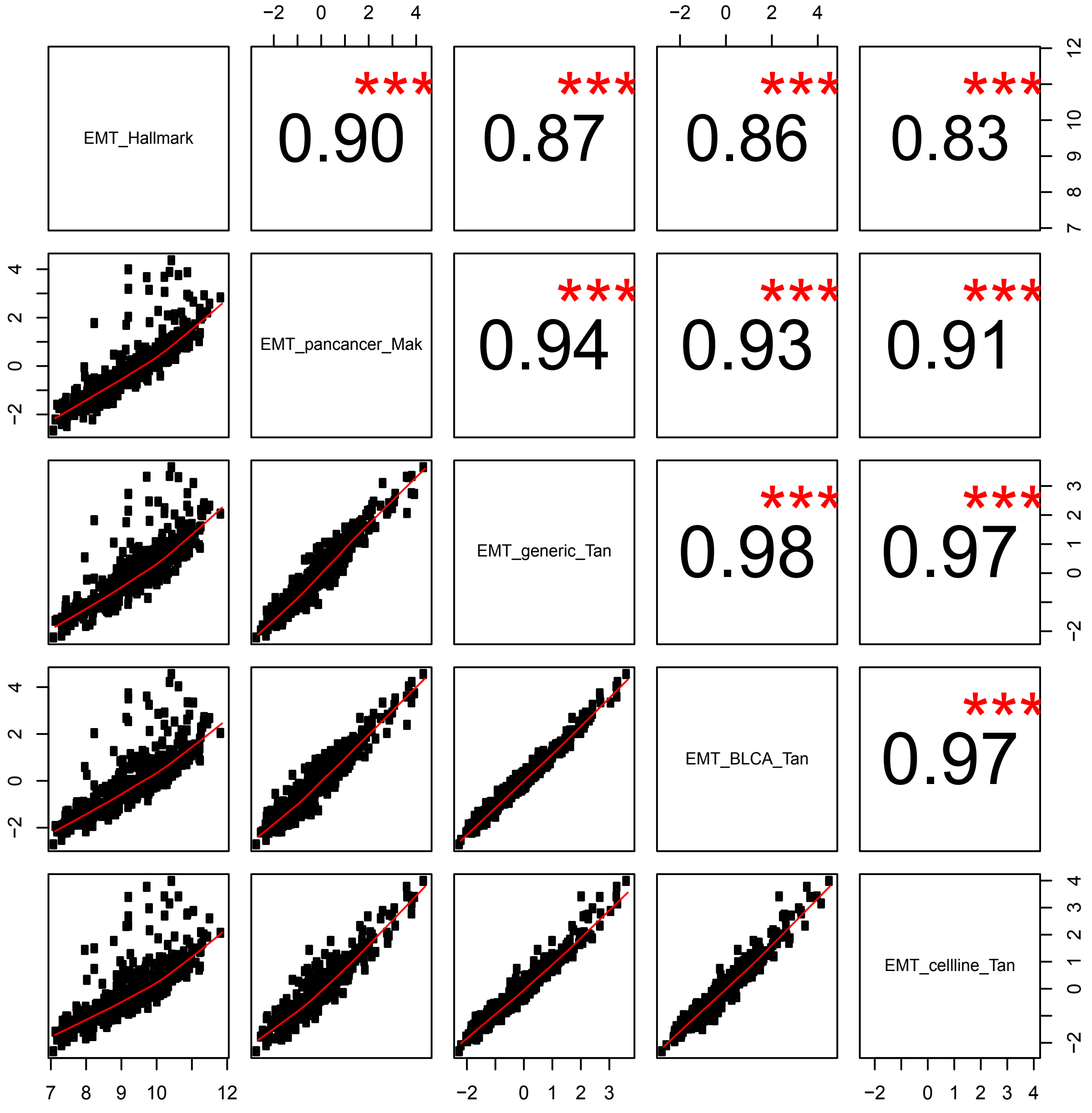

Pair-wise correlation of EMT scores estimated by different sets of EMT markers

Supplementary Fig. 2

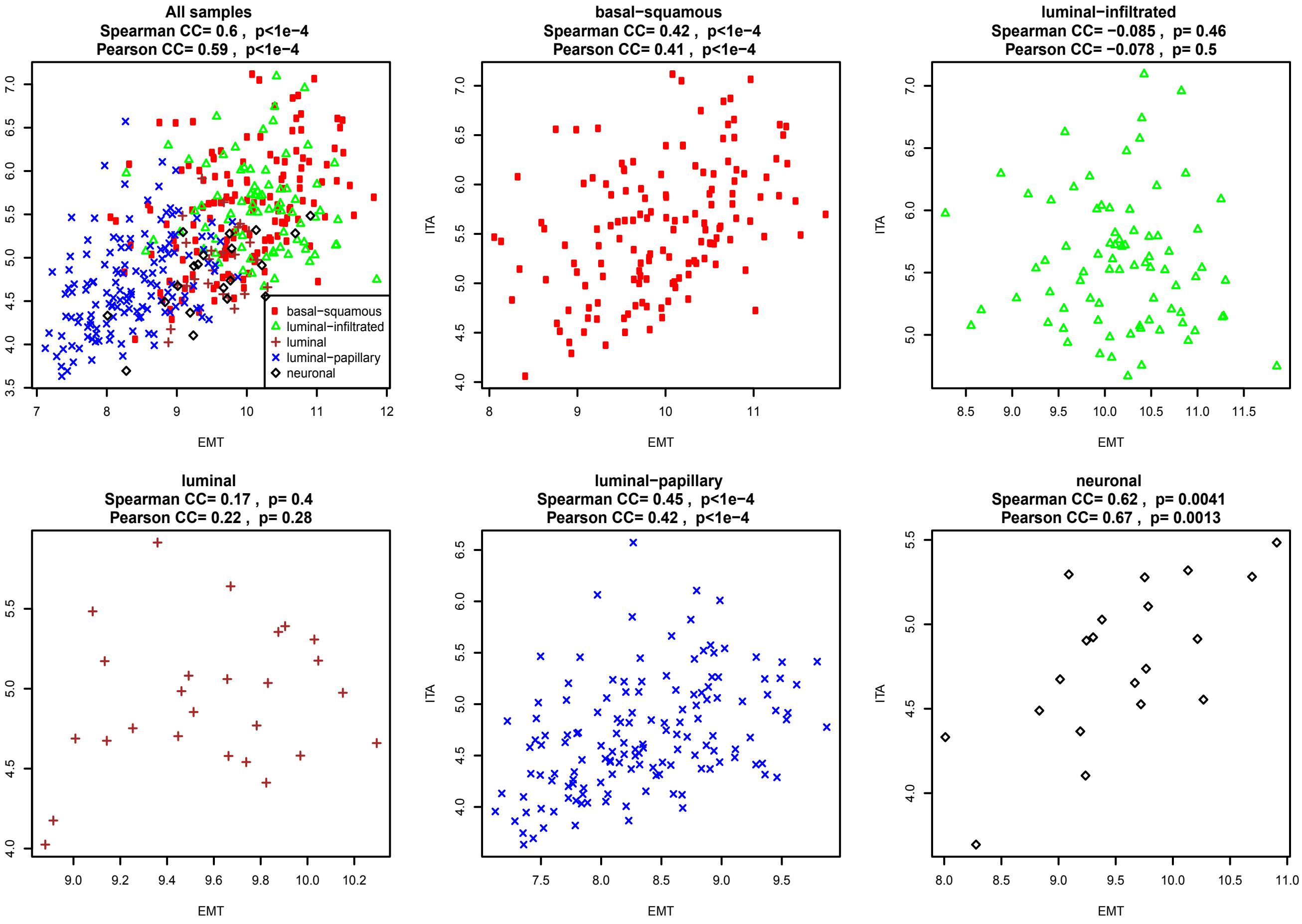

Plot of EMT vs ITA within different subtypes of UC in TCGA.

Supplementary Fig. 3

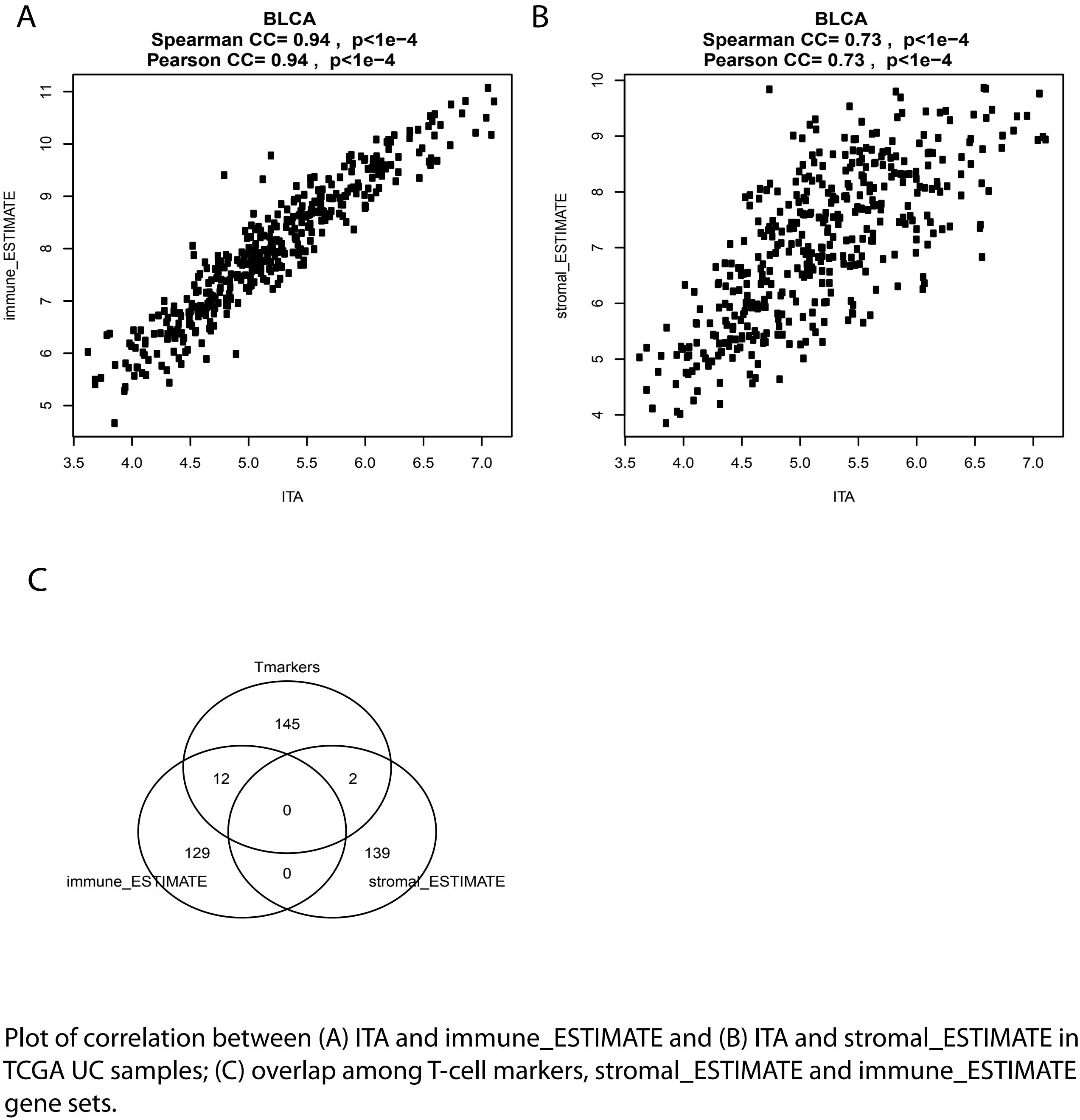

Plot of correlation between (A) ITA and immune_ESTIMATE and (B) ITA and stromal_ESTIMATE in TCGA UC samples; (C) overlap among T-cell markers, stromal_ESTIMATE and immune_ESTIMATE gene sets.

Supplementary Fig. 4

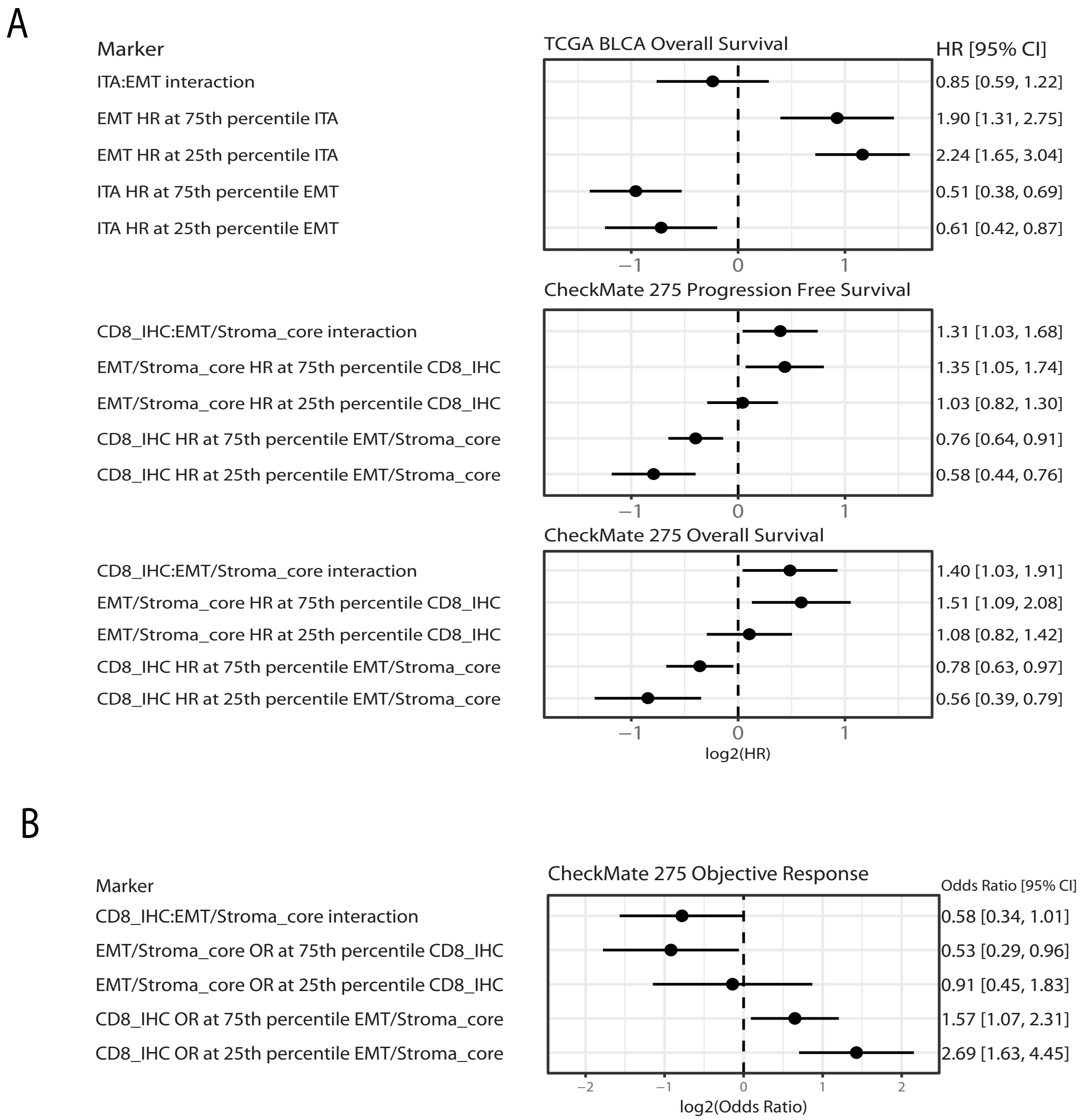

(A) Hazard Ratio estimates for OS in TCGA subjects and for PFS and OS in CCheckMate 275 subjects. Plotting symbols give point estimates of log2(HR); horizontal bars give 95% confidence intervals. HRs were scaled to compare 75th and 25th percentiles of biomarker scores. For the interaction terms, we plotted log2(HR for a biomarker at the 75th percentile of the other biomarker) - log2(HR for that biomarker at the 25th percentile of the other biomarker), which gives log2 of the ratio of HRs. (B) Odds Ratio (OR) estimates for objective response in CheckMate 275 subjects. Plotting symbols give point estimates of log2(OR); horizontal bars give 95% confidence intervals. ORs were scaled to compare 75th and 25th percentiles of biomarker scores. For the interaction terms, we plotted log2(OR for a biomarker at the 75th percentile of the other biomarker) - log2(OR for that biomarker at the 25th percentile of the other biomarker), which gives log2 of the ratio of ORs.

Supplementary Fig. 5

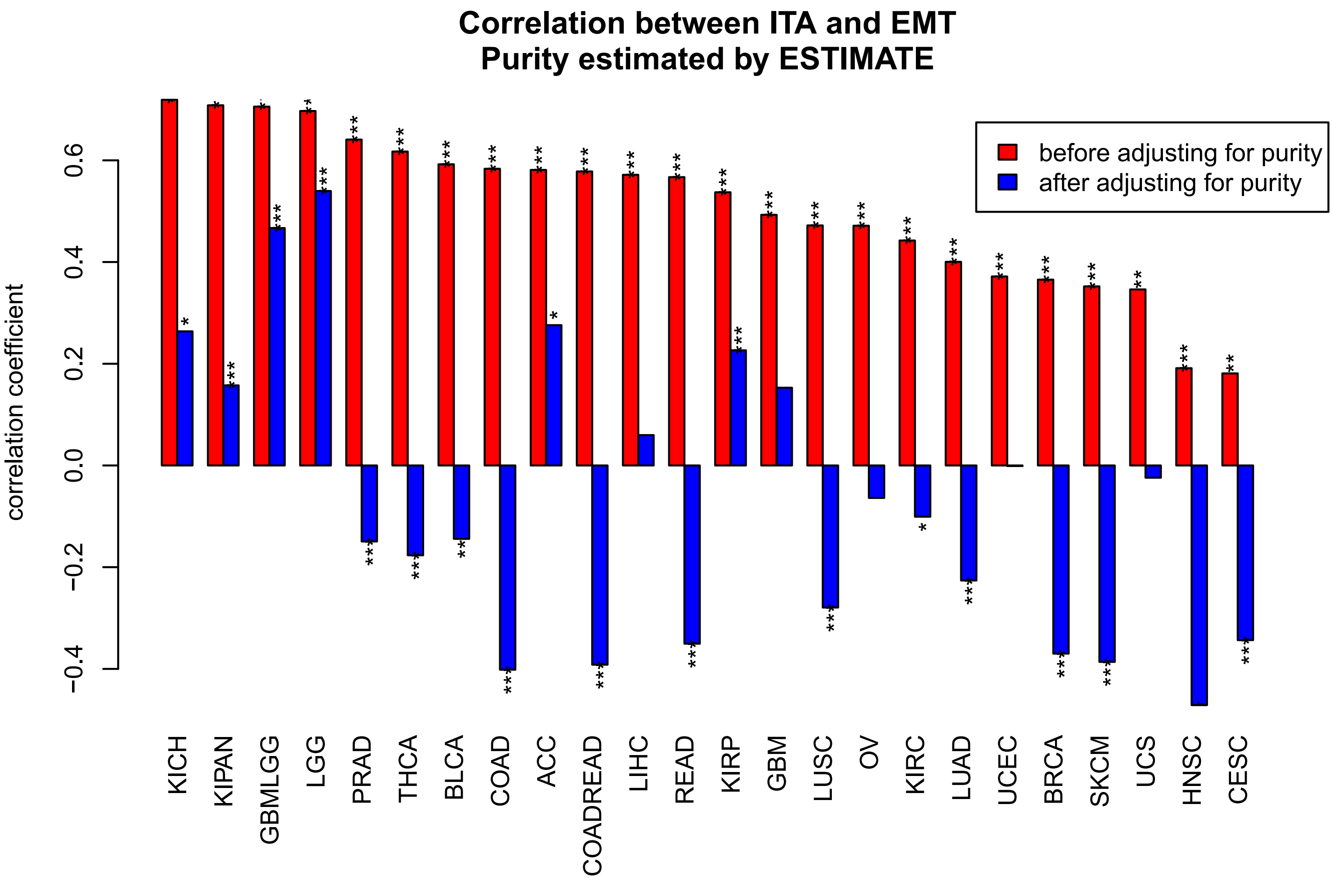

Pan-cancer analysis of correlation coefficients (Pearson's) between ITA and EMT before and after adjusting for purity estimated by ESTIMATE method (*0.1<p<0.05. **0.001<p<0.01, ***p<0.001 by Pearson's correlation test).

Supplementary Fig. 6

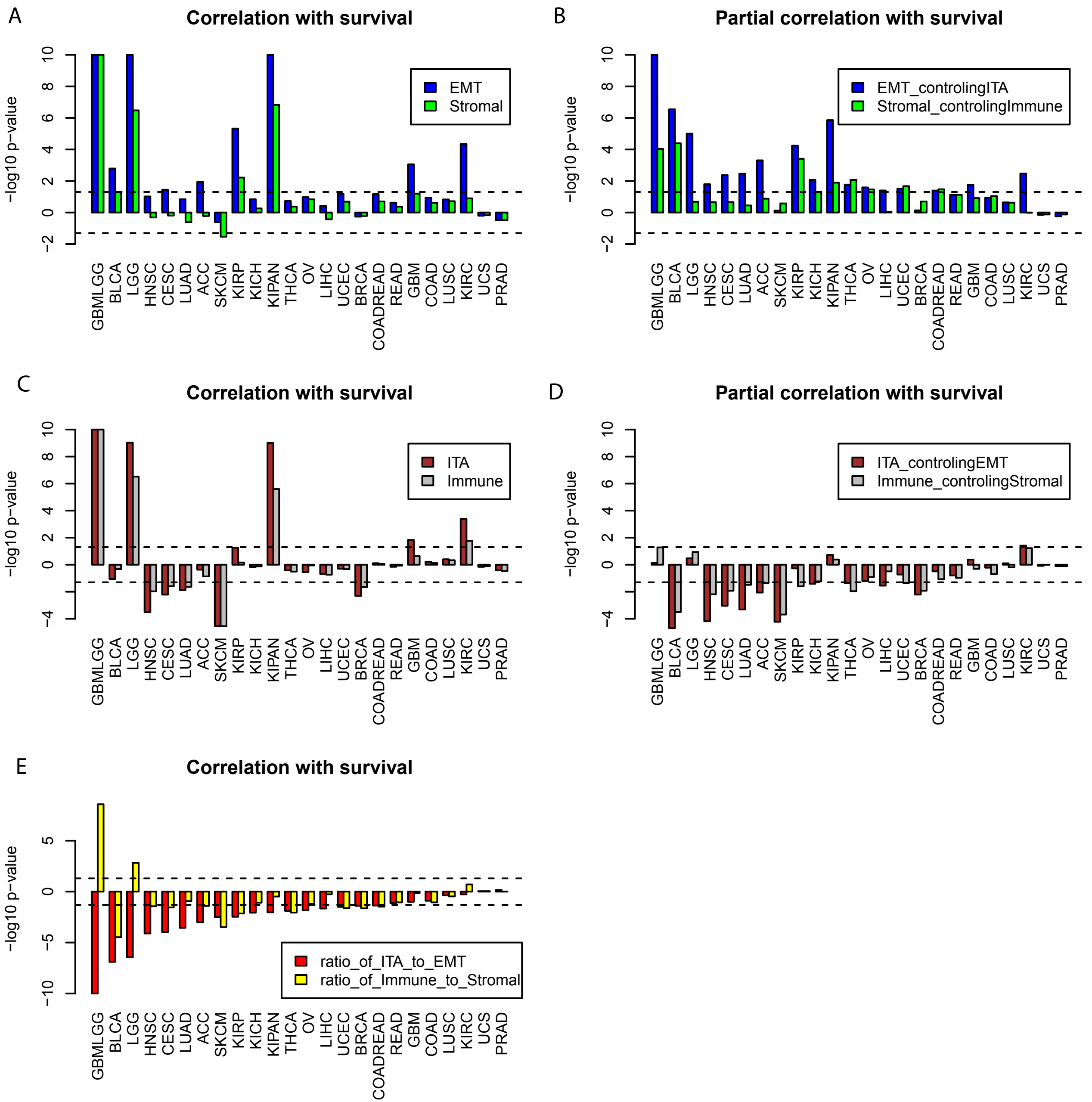

Pan-cancer analysis of the prognostic significance of (A) EMT and stromal gene signatures, (B) EMT conditional on ITA, (C) ITA, (D) ITA conditional on EMT, (E) and ITA:EMT ratio. Univariate Cox regression model was used in (A), (C) and (E) and bivariate Cox-regression model was used in (B) and (D). Y-axis shows -log10 p-value of Wald's test (those associated with worse survival were assigned log10 p-value instead).

Supplementary Fig. 7

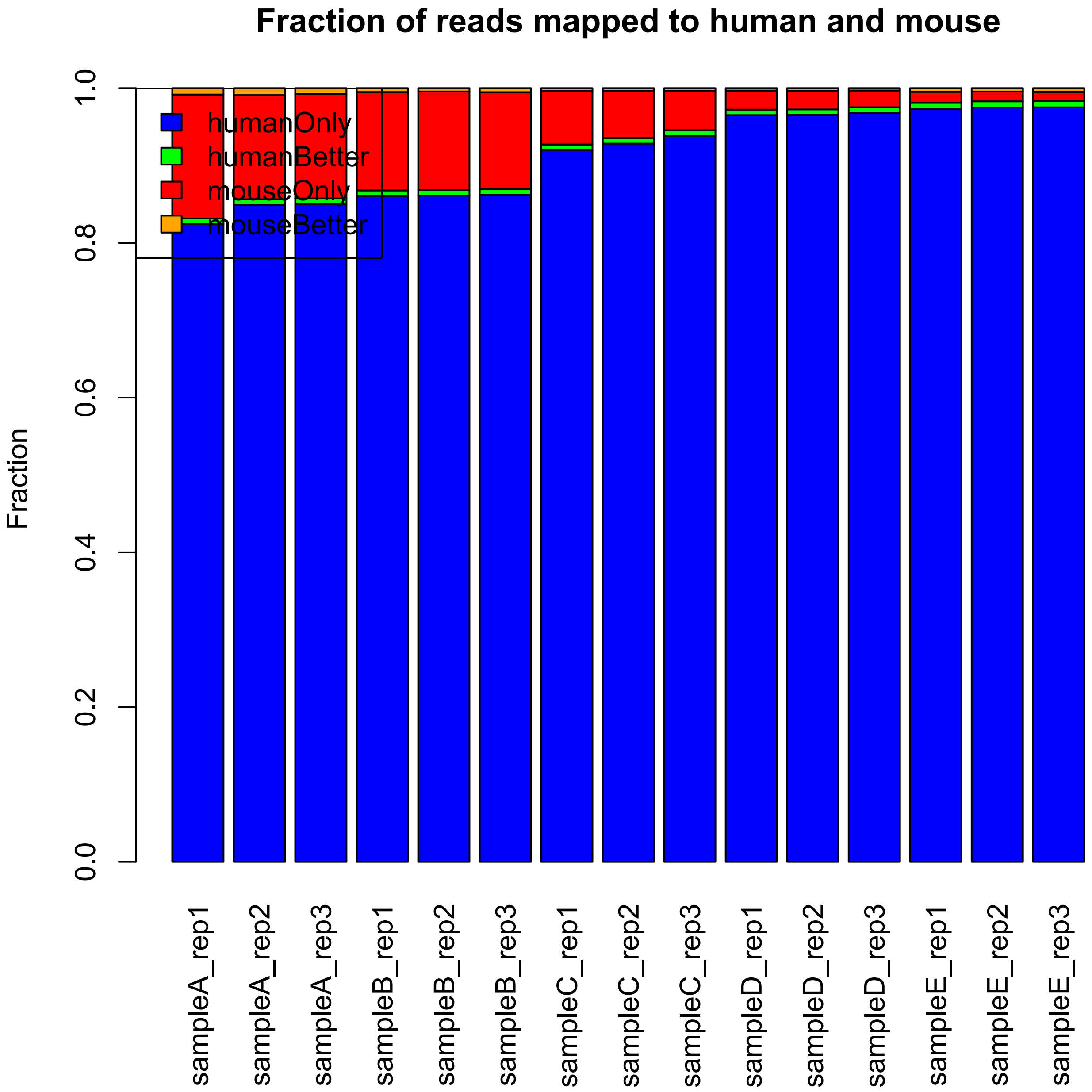

Fraction of RNA reads mapped to human and mouse for each of the five PDX models (3 replicates for each model).

Supplementary Fig. 8

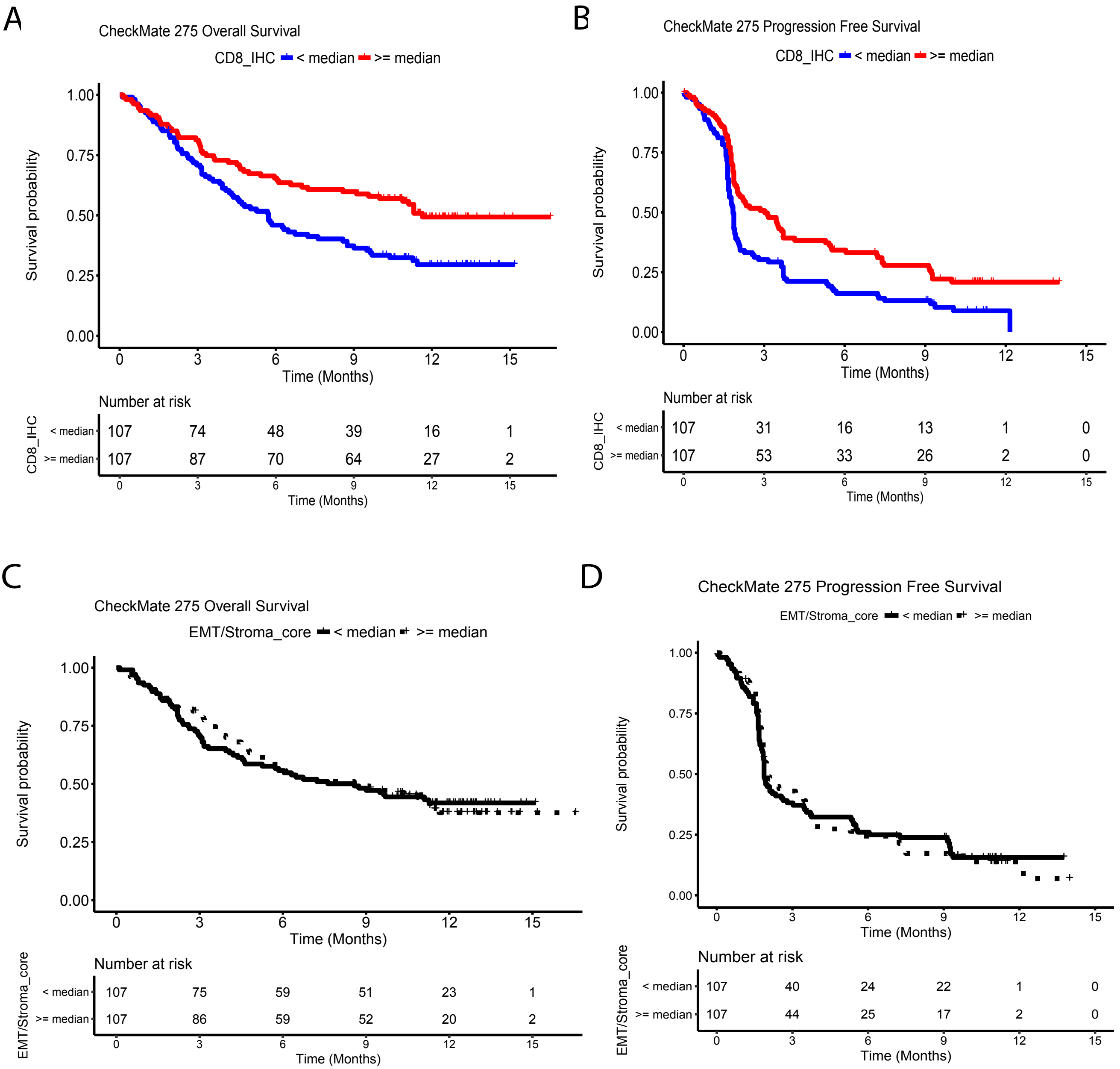

OS and PFS curves in patients in the CheckMate 275 biomarker cohort, stratified according to the CH8_IHC scores (A,B) or EMT/Stroma_core scores (C,D).

Supplementary Fig. 9

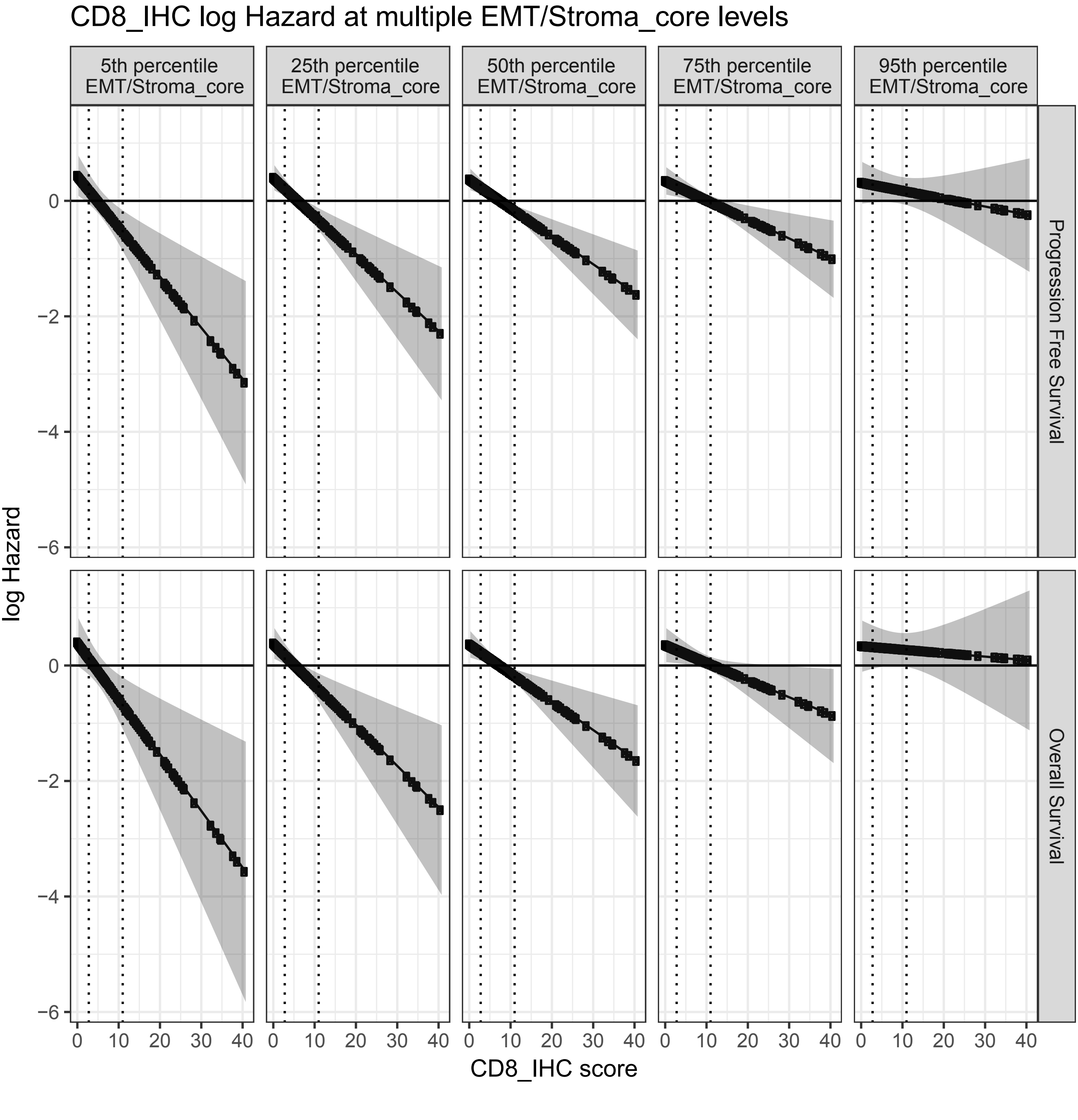

Predicted log hazard curves demonstrating the association between PFS or OS and CD8 IHC at various (5th, 25th, 50th, 75th and 95th percentile) EMT/Stroma_core levels, using CD8_IHC + EMT/Stroma_core + CD8_IHC:EMT/Stroma_core Cox model. CD8 strongly associated with PFS or OS at low and medium core_EMT values, but loses its association at the high core_EMT levels. Shaded areas show pointwise 95% confidence intervals.

Supplementary Fig. 10

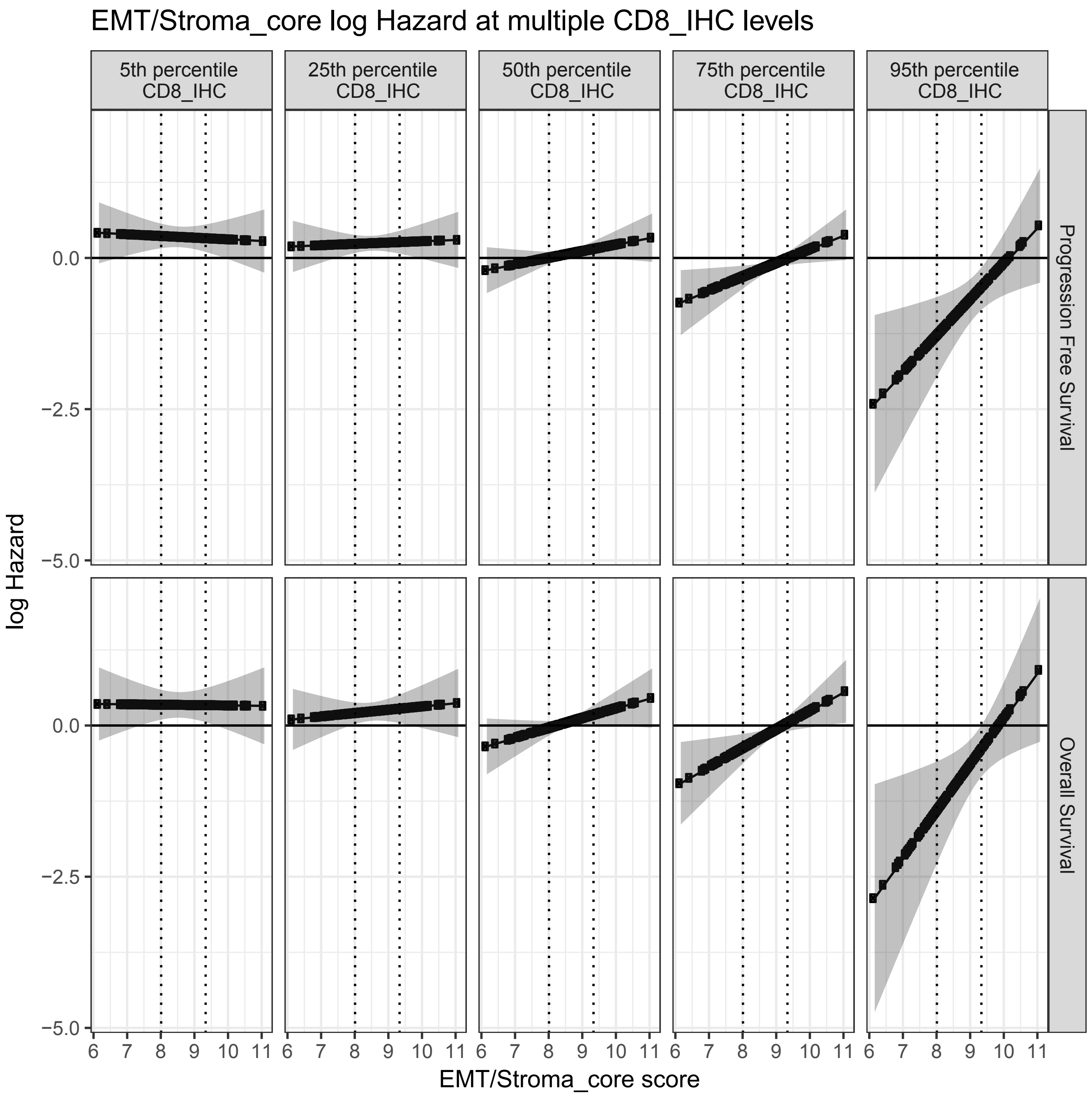

Predicted log hazard curves demonstrating the association between PFS or OS and EMT/Stroma_core at various (5th, 25th, 50th, 75th and 95th percentile) CD8_IHC levels, using CD8_IHC + EMT/Stroma_core + CD8_IHC:EMT/Stroma_core Cox model. EMT/Stroma_core strongly associated with PFS or OS at high CD8_IHC values, but loses its association at the low CD8_IHC levels. Shaded areas show pointwise 95% confidence intervals.

Supplementary Fig. 11

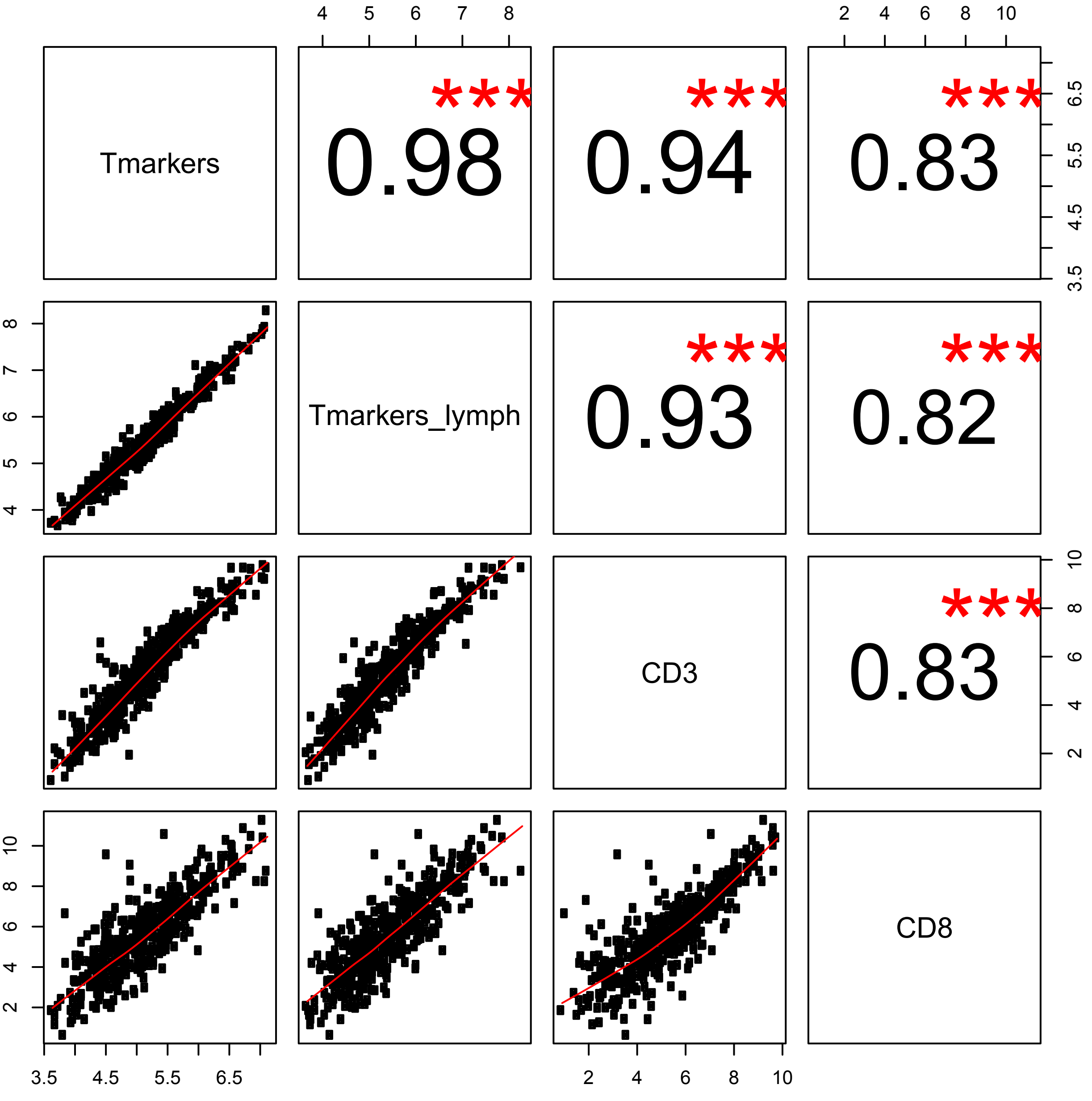

Pair-wise correlation of ITA estimated by different sets of T-cell markers.

Supplementary References

1. Barretina, J. *et al.* The Cancer Cell Line Encyclopedia enables predictive modelling of anticancer drug sensitivity. *Nature* **483,** 603–7 (2012).

2. Mak, M. P. *et al.* A Patient-Derived, Pan-Cancer EMT Signature Identifies Global Molecular Alterations and Immune Target Enrichment Following Epithelial-to-Mesenchymal Transition. *Clin. Cancer Res.* **22,** 609–20 (2016).

3. Tan, T. Z. *et al.* Epithelial-mesenchymal transition spectrum quantification and its efficacy in deciphering survival and drug responses of cancer patients. *EMBO Mol. Med.* **6,** 1279–1293 (2014).

4. Clopper, C. J. & Pearson, E. S. The Use of Confidence or Fiducial Limits Illustrated in the Case of the Binomial. *Biometrika* **26,** 404–413 (1934).
